# Genenames.org: the HGNC resources in 2015

**DOI:** 10.1093/nar/gku1071

**Published:** 2014-10-31

**Authors:** Kristian A. Gray, Bethan Yates, Ruth L. Seal, Mathew W. Wright, Elspeth A. Bruford

**Affiliations:** HUGO Gene Nomenclature Committee, European Molecular Biology Laboratory, European Bioinformatics Institute, Wellcome Trust Genome Campus, Hinxton, Cambridge, CB10 1SD, UK

## Abstract

The HUGO Gene Nomenclature Committee (HGNC) based at the European Bioinformatics Institute (EMBL-EBI) assigns unique symbols and names to human genes. To date the HGNC have assigned over 39 000 gene names and, representing an increase of over 5000 entries in the past two years. As well as increasing the size of our database, we have continued redesigning our website http://www.genenames.org and have modified, updated and improved many aspects of the site including a faster and more powerful search, a vastly improved HCOP tool and a REST service to increase the number of ways users can retrieve our data. This article provides an overview of our current online data and resources, and highlights the changes we have made in recent years.

## INTRODUCTION

The HUGO Gene Nomenclature Committee (HGNC) is responsible for providing unique and informative nomenclature for all genes within the human genome (1). The majority of our data are manually curated, and we collaborate with researchers working on particular genes and gene families to create names that are acceptable to researchers in the field. HGNC symbols and names are seen as a standard and used in all the major databases that concentrate on human genes and proteins, such as Ensembl (2), UniProt (3), NCBI Gene (4), ENA/GenBank/DDBJ (5–7), Vega (8), GeneCards (9) and the UCSC genome browser (10), as well as disease and phenotype resources including Decipher (11), OMIM (12), Locus Reference Genomics (LRG)(13), ClinVar (14) and GeneTests (15). By working closely with other species’ nomenclature groups such as mouse (16), rat (17) and zebrafish (18) we ensure that orthologous genes are assigned equivalent symbols where possible.

We also maintain a website http://www.genenames.org that provides a public access portal without any restrictions to the data, with tools to search, download and discover genes, gene families and possible orthologs. More information about our website and applications will appear later in this article.

## DATA

As of the beginning of August 2014, we have 39 135 active entries within our database, with 19 064 of the entries being protein coding genes. Comparing the number of protein coding genes with the figure quoted in our last Nucleic Acids Research database issue article (19) of 19 027, it is clear that the number is plateauing at the low 19 000 mark. In June 2014, Ezkurdia and co-workers released an article (20) in which they describe comparing peptide mass spectroscopy results with GENCODE (21) data and concluded that the number of protein coding genes will approximately be 19 000 within human. The CCDS project (discussed below), whose remit is to identify a core set of protein coding regions, have assigned CCDS IDs to 18 681 genes as of August 2014. Taking these two pieces of information into account, we believe that we have an almost complete set of protein coding genes for the human genome.

This year (2014) the HGNC have become members of the consensus coding sequence (CCDS) project (22), which aims to develop a set of high-quality gene annotations for human and mouse protein-coding genes. The project is an international collaboration between the Ensembl (2), HAVANA (8), NCBI RefSeq (23), MGI (16) and HGNC teams. NCBI provides automatic and manually curated RefSeq annotations, and Ensembl provides an annotation set that includes Ensembl gene model predictions and manual gene annotations by HAVANA. Protein-coding annotations with matching genomic coordinates that pass a set of stringent quality control tests are assigned a unique CCDS identifier (ID). CCDS IDs that are marked for changes of any kind undergo a review process that includes annotators from RefSeq and HAVANA, and curators from the HGNC for human genes and from MGI for mouse genes.

The largest area of growth within our Symbol Reports is currently long non-coding RNA (lncRNA) genes, with entries having increased by 69% since 2012 (19). For a full review of how we name lncRNA genes please see our recent article (24). The number of approved gene symbols for pseudogenes is also rising rapidly with entries having increased by 50% since 2012 (19). Our pseudogene locus type now includes pseudogenes of RNA genes such as *RN7SKP1* (RNA, 7SK small nuclear pseudogene 1) and also functional pseudogenes, which we define as transcribed pseudogenes that have been reported to have a functional role in a publication. These can be identified in our website by inclusion of ‘(functional)’ at the end of the gene name, e.g. the full name for *MYLKP1* is ‘myosin light chain kinase pseudogene 1 (functional)’. A link to the source publication is included in each relevant Symbol Report, and we maintain a webpage where all functional pseudogenes can be viewed (http://www.genenames.org/functional-pseudogenes). Another area of growth within our database has been the increase in the number of gene families, something we commented on in our last article (19). To date, we have 588 families with 51% of the protein coding genes within our database associated to at least one family.

The human genome build is no longer just the ‘golden path’. To adequately represent complex structural variations, the Genome Reference Consortium includes alternative loci within their build of the human genome (25). The HGNC aims to name all structural variants annotated on alternative reference loci as described in (26). These genes can be identified within our Symbol Reports by the Chromosomal Location field, which displays the location plus the term ‘alternate reference locus’ and can be viewed in full using the table provided on our Statistics and Downloads page labelled ‘Alternative Loci Statistics’.

## WEBSITE

Our website http://www.genenames.org is the public access portal to our data, and since the new design release in May 2011 we have built upon the site, updating and improving many sections including our search and HCOP tool, as well as redesigning the homepage. We have moved the karyotype image to the statistics and downloads page, and replaced it with a word cloud of common root symbols. Each root symbol within the word cloud is a link that retrieves a search result of genes containing the root symbol. Another addition to the design is a new search bar found in the masthead of every page. By default, the search bar will search the symbol reports, but users have the option of searching the whole site by selecting ‘site search’ in the drop down.

The site and database now run from two offsite data centres with multilayer redundancy using virtual machines. Our user requests are now load balanced between data centres and servers, providing quicker response times during peak times. This infrastructure also allows maintenance without interruption of service, by taking one data centre offline to work on while the other continues to serve content. As well as the infrastructure changing, a number of our pages and applications have been refactored or replaced, and so have the URLs (see http://www.genenames.org/news/new-features-and-changes#URL_changes_-_2014--01--08). Below is a brief tour of the changes and additions we have made to the site within the last two years.

### Symbol reports

The symbol reports are our most visited pages as this is our main interface to the manually curated nomenclature and external database links. Below, we briefly outline the key updates we have made to the symbol reports.

#### Homologs

This section has been reformatted into a table structure to show the rat and mouse homologs of the human gene. The table is split into three columns: organism, symbol and database which displays the gene ID for that species’ model organism database. Currently we only show mouse and rat homologs but this may change in the future. In the past we only showed one ortholog per species but now we show multiple paralogous genes per species which better represents homology across the species.

#### New links

‘Protein resources’ include a new link to PDBe (27) in addition to the links to UniProt and Interpro (28). This means we now have links to a resource that describes the protein in general, the domains it contains and the 3D structure of the protein all linked by the protein accession. A new clinical resource link that we have added is to the Genetics Testing Registry (GTR) (29). The GTR is a repository of voluntary submissions of genetic testing methodologies and purpose. We have also added a new ‘*Other databases*’ link to BioGPS (30) which is a gene annotation portal that allows the user to ‘plugin’ resources into the application for a gene. By default, the BioGPS link will display a graphical representation of gene expression based on GeneAtlas U133A, gcrma data set.

#### References

**Figure 1.**
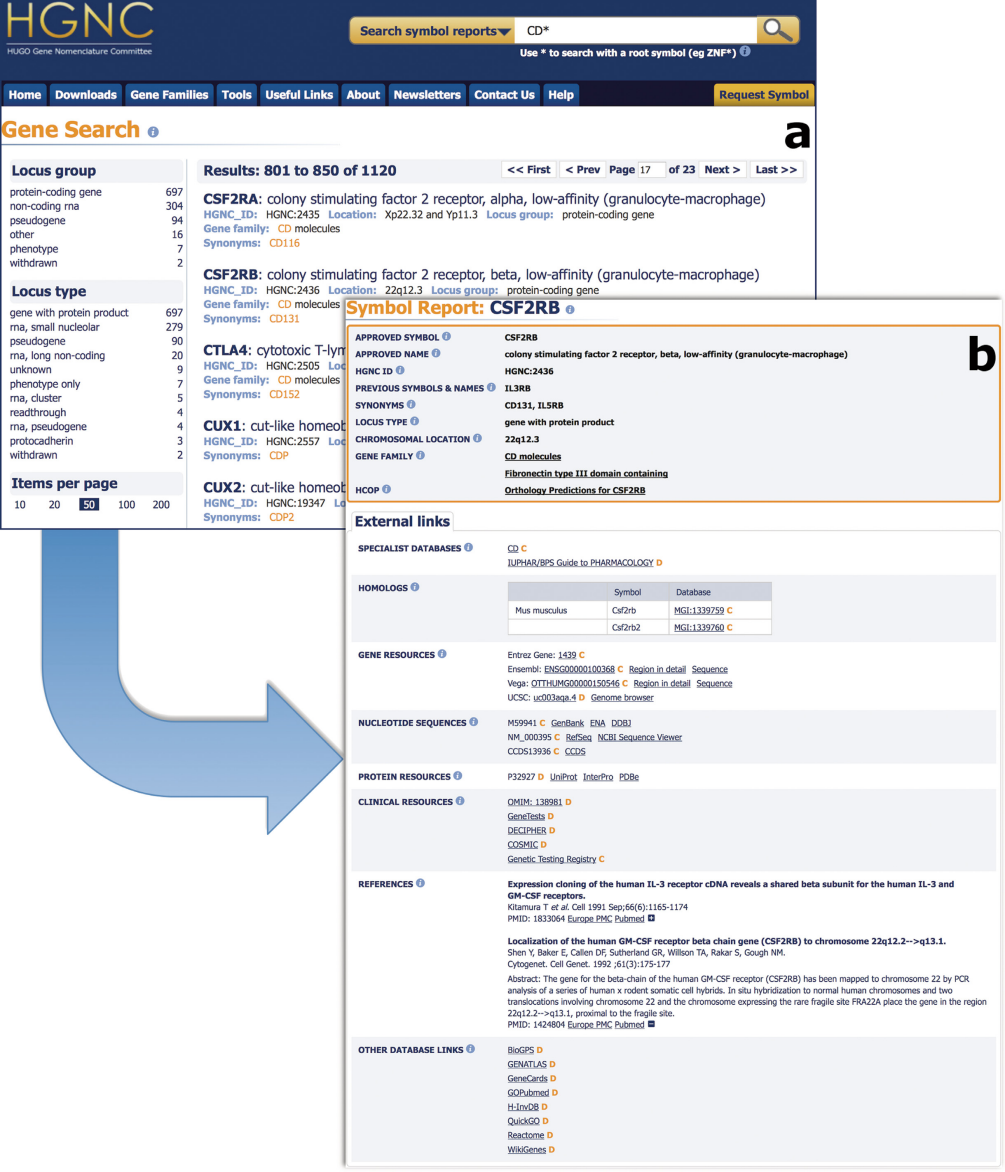
The new search box located within the masthead activates the search and returns the results as seen above in section ‘**a**’. The results are faceted as seen on the left allowing the user to filter the returned results by locus type and group. The results display some of the core HGNC data such as symbol, name and ID. Each result also highlights the field(s) the term matches. Section ‘**b**’ displays a typical symbol report showing the HGNC core data fields within the shaded box. New additions to the core data are the HCOP and gene families links that have migrated from the external links section below. A slightly changed format to the external links section can be seen including our improved references section. The first reference is the default view while the second displays the full author list and abstract as would be displayed once the plus sign is clicked. Clicking on the minus will return the reference back to the default. Another addition is the support for multiple mouse and rat homologs per human gene within our symbol reports as seen above.

The references section of the ‘external links’ section has changed considerably. In the past we displayed the PMID number, a link to PubMed (31) and a link to CiteXplore which has now become Europe PubMed Central (Europe PMC) (http://europepmc.org/). What we display now by default is the title, article reference, PMID number and links to both Europe PMC and PubMed. Next to the links to PubMed we have added a plus sign which when clicked, alters the reference to include the full author list and abstract. This is all done with an AJAX request to the Europe PMC RESTful web service, retrieving the information needed by PMID number and rendering the information within the symbol report page (Figure 1b).

### New faster and smarter gene search

In January 2014 we replaced the ‘quick search’ application with a Solr search engine to search symbol reports. Solr is an open source search engine, built upon apache lucene, that many of the world's largest websites have adopted since it offers very quick full-text searches. Solr servers are also highly scalable and offer efficient replication to other search servers if needed.

Adding a query term into the input box within the masthead and clicking on the spyglass activates a search. By default, the search term is used for a full-text search over all the indexed fields. Our search also allows users to match records based on a query pattern using the wildcard and logical operators with OR being the default operator. Double quoting the search query term will tell the search to treat the quoted block as one term. Users can search against specific indexed fields by using a colon between the indexed field key and the term.

The results page contains two columns. On the left we utilise Solr's ability to provide facets. The results are faceted by locus group and locus type with counts of the number of reports that fit the facets (Figure 1a). Also within this column users can change the default number of results displayed per page from 10 up to 200. In the right column we list the results displayed primarily in order of relevance and then alphanumerically on symbol. The first line of a ‘hit’ contains the approved gene symbol and the approved gene name as defined by the HGNC. On the second row we display the HGNC ID, the chromosome location (if specified) and the locus group to which the entry belongs. The remaining row reports the field type the keyword/ID matches and highlights the query term within the field. If the number of results is greater than the number of displayed items on a page, a pager will appear, allowing users to navigate through the results. For a list of indexed fields and more information on the advanced features of the search, please see http://www.genenames.org/help/search.

### Downloads

As well as our pre-existing BioMart and custom downloads applications we have introduced a new REST service. This is a new addition to the site allowing users to search/query our database and retrieve data in an XML or JSON format from within a calling script or program. The REST service is built upon our Solr search engine and has its own URL of http://rest.genenames.org. The service has three main requests, ‘info’, ‘search’ and ‘fetch’. The info request is the simplest in that the URL is http://rest.genenames.org/info without any other parameters. The info request notifies the user what fields can be queried and what information is stored. Info also returns exactly when the data were modified and the total number of records including records with an ‘Entry Withdrawn’ status. Changes to the service such as new or removed fields will be seen using the info request. The other two requests to the REST service are search and fetch. Since the server is built upon Solr, searching for records is far quicker than fetching all stored fields; therefore for a large query the user should request a search and then fetch individual records. The search and fetch requests can utilise the same indexed fields as mentioned within the search section of this article, but the search request will only return the HGNC ID, symbol and score of the search. By default, the search request will search all indexed fields if no field is provided (e.g http://rest.genenames.org/search/ZNF3). If a specific field should be searched then the field should come between the request type and the term (e.g http://rest.genenames.org/search/symbol/ZNF3). The full arsenal of complex query methods is available to the search request such as phrases, wildcards and logical operators. The fetch method requires the user to add a searchable field and the query term to the URL (e.g http://rest.genenames.org/fetch/hgnc_id/HGNC:1097) and will not accept complex queries like wildcards. Fetch will however return all stored fields as described within an info request result and therefore the entire gene symbol report in XML or JSON format. For detailed information on how to use our REST service please visit http://rest.genenames.org.

### Multi-symbol Checker

This tool has replaced the ‘list search’ tool but contains the same functionality as the old application and introduces a sortable results table, filtering by match type, and an increase in speed for large symbol lists. We have redesigned the form for the application to take full advantage of the width of the page and reduce the overall real estate size of the form. Lists of gene symbols can be added to the text box or uploaded to the site within a file. Comma, space, new line or a mixture of the three can separate the symbols within the file or text box. As well as returning a sortable table of results the user can request for the results to be returned as tab delimited plain text. As the name suggests the main functionality of this tool is to check a batch of symbols from the user to see if these symbols are still current; however it can also be used as a bulk symbol search to browse the symbol records within our database for a given set.

### HCOP

A new and improved version of our HGNC Comparison of Orthology Predictions (32,33) tool has been released. HCOP currently collates orthology assertions from eggNOG (34), Ensembl Compara (2), HGNC, HomoloGene (31), InParanoid (35), OMA (36), OrthoDB (37), OrthoMCL (38), PANTHER (39), PhylomeDB (40), Treefam (41) and ZFIN (18) into a single tool, enabling comparison of these data to identify a consensus orthology prediction for a specified human gene or set of genes. Data are integrated using pre-existing mappings from the orthology resources to link between model organism databases, NCBI Gene and Ensembl. An indication of the reliability of a prediction is provided by the number of orthology databases which concur. HCOP was originally designed to show orthology predictions between human and mouse, but has since been expanded to include data from chimp, macaque, rat, dog, horse, cow, pig, opossum, platypus, chicken, anole lizard, xenopus, zebrafish, *C. elegans*, fruitfly and *S. cerevisiae*, meaning that there are now 18 genomes available for comparison in HCOP.

The updated version of HCOP involved a complete rewrite of the pipeline used to combine the data from each of the orthology sources. The data behind HCOP are stored in a MySQL database to allow for rapid querying. Each orthology assignment is stored as a pair of genes with a list of associated databases that support that assertion. The data are updated weekly by running a pipeline developed using the eHive (42) production system from Ensembl. In addition to the orthology data we also import gene data from HGNC, MGI, RGD (17), Xenbase (43) and ZFIN to ensure that we have the current approved gene symbols, names, locus types and location information from the appropriate nomenclature or model organism database. For those species without an official nomenclature committee or where a nomenclature committee exists but gene data are not available in a form our pipeline can utilise, we take this information from the NCBI Gene database, or from Ensembl if the gene in question cannot be mapped to an NCBI Gene identifier.

**Figure 2.**
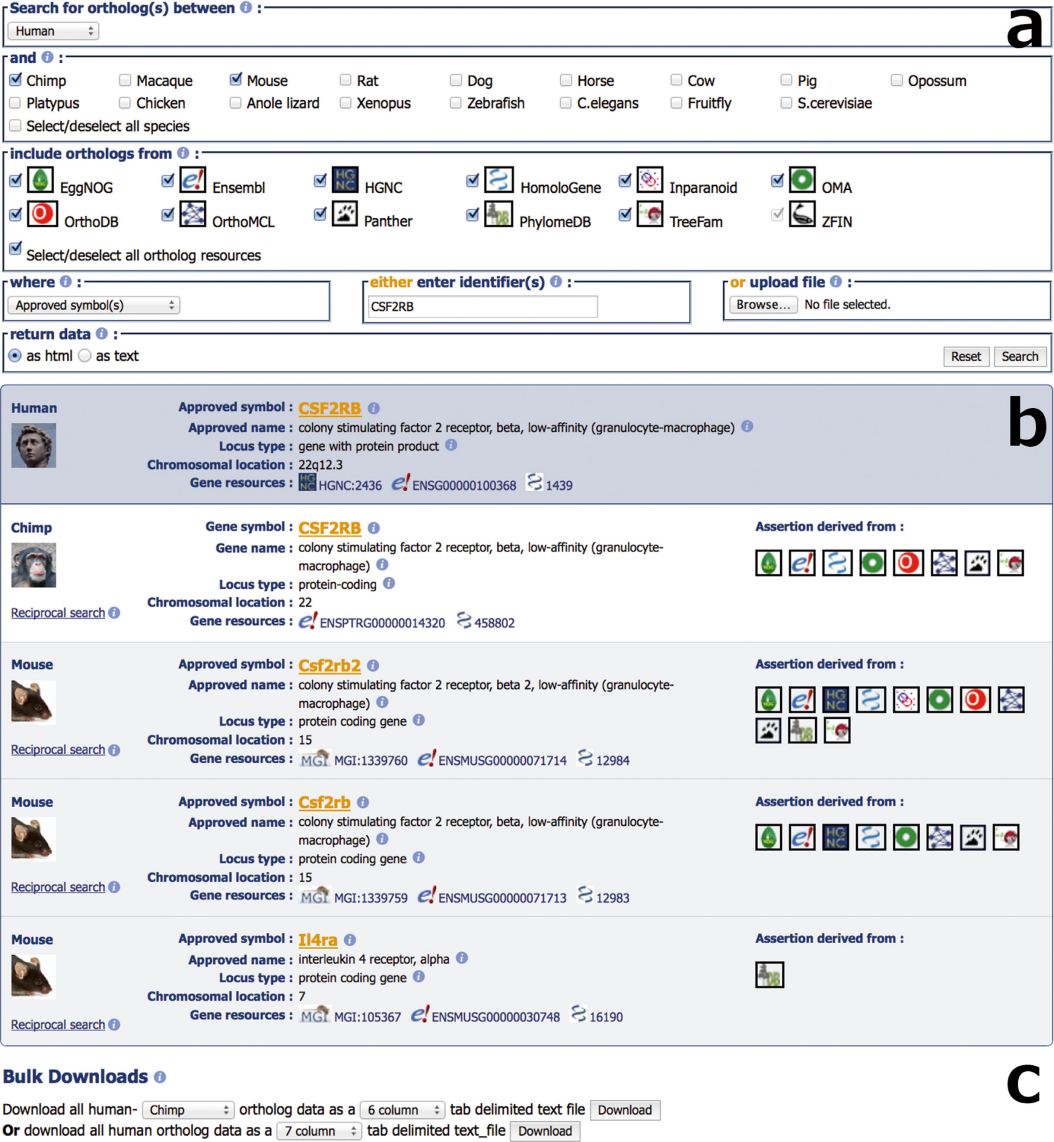
Section ‘**a**’ shows the updated HCOP search form. Users select a primary species and one or more species that they wish to identify orthologs in. They then select the ortholog resources they wish to include in the search, and the type of search term, e.g. approved symbol, or database identifier, that they are providing. A single search term or list of search terms may be pasted into a text box, or uploaded as a file to be used to run the search. Users can optionally see the results in HTML or plain text format. Section ‘**b**’ displays an example result panel from HCOP. In this case orthologs between the human gene *CSF2RB* in chimp and mouse were requested. Information about the query gene appears in the blue section at the top of the results panel, with each ortholog identified having its own section below this. For both the query and ortholog genes we provide basic information about the gene as well as links to the gene in other resources. Each ortholog section has an additional column labelled ‘Assertion derived from’ that contains a set of icons that represent the orthology sources that support this assignment. Section ‘**c**’ is the bulk downloads section of the HCOP form. This allows users to download pre-computed files of HCOP data from our FTP site, providing fast access to all the data stored in HCOP.

The HCOP user interface (see Figure 2a) has undergone a redesign to improve its usability while still retaining the functionality of the original version. For species with either a model organism or nomenclature database you can search using an approved gene symbol, an approved gene name or the gene identifier from that database. Orthology assertions can also be obtained for a gene by searching with its Ensembl gene identifier or its NCBI Gene identifier. In the latest version of HCOP you can now specify which species and orthology sources you wish to see represented in the results. The consensus orthology assertions for multiple genes can be viewed simultaneously by searching with a list of query terms.

The display of HCOP results (see Figure 2b) has changed significantly. A scrollable results panel is displayed for each query term that produces a result. At the top of the results panel in the blue section, you will see data relating to the query term you supplied. Below, this you will see a section for each ortholog that has been returned. For both the query and orthologs you should see a gene symbol, a gene name, a locus type, a chromosomal location and a set of gene resources. The gene symbol and gene name will be prefixed with either ‘Approved’ to indicate that it was assigned by a nomenclature committee or ‘Gene’ to indicate that it is not an approved symbol or name. A non-approved symbol may in some cases be listed as ‘Unknown’ when no symbol has been assigned to that gene. Hovering your mouse over the information icons in the results panel will tell you where the data originate. For each orthologous gene you will also see a section labelled ‘Assertion derived from:’ that contains a series of icons. These icons represent the sources that support this orthology assignment. Clicking on the icon will take you to the entry for that gene in the orthology database, while hovering over the icon will give you the full name of the ortholog data source.

In addition to the new user interface, we also provide pre-calculated files of HCOP data that can be downloaded from our FTP site or via the ‘Bulk downloads’ section (see Figure 2c) of the HCOP page. You have the option of getting a file containing human and ortholog data from a single species, or human and ortholog data from all HCOP species in a single file. For the human—single ortholog species files the ‘6 Column’ output returns the raw assertions, Ensembl gene IDs and Entrez Gene IDs for human and one other species, while the ‘15 Column’ output includes additional information such as the chromosomal location, accession numbers and, where possible, references the approved gene nomenclature. The files containing all species’ ortholog data have an additional column at the start giving the taxon id for each species.

## FUTURE DIRECTIONS

We will continue to improve and add functionality to our website, in particular to the gene search application and to our REST service. One such planned addition is the ability to search/retrieve genes by chromosome. External links within symbol reports will continuously be reviewed and new links to noteworthy resources added, such as ClinVar and GeneReviews (http://www.genereviews.org).

A major change to the site in the near future is a completely new gene families section. As mentioned earlier in the article, our gene families’ data are growing in size and complexity. Some of our large gene families contain hierarchies such as ‘Class A GPCRs, rhodopsin-type’ which has subset families (e.g ‘Olfactory receptors’) and is itself a subset of the ‘G protein-coupled receptors’ family. Our new gene family pages will display the relationships between subsets better and allow users to browse through the hierarchy. Many families will contain a brief description and may contain a graphical display of the protein domains of a typical family member. With a better built in hierarchy within the new pages we can also provide a much improved way of downloading gene families as data sets, allowing people to choose between downloading one subset family or the entire family hierarchy. The new gene families will be searchable via our Solr search engine, and the index page for the families will have a searchable and sortable, paged table all of which we hope will improve our users’ experience.

In addition the HGNC have recently been funded to expand our remit to naming genes across all vertebrate species that do not have an official gene nomenclature committee. The naming will be based on the names of orthologous and paralogous human genes. These pan-vertebrate data will be accessed from a new website, with reciprocal linking to the human homologs at the current HGNC website (www.genenames.org).
